# A Community-Wide Intervention Trial for Preventing and Reducing Frailty Among Older Adults Living in Metropolitan Areas: Design and Baseline Survey for a Study Integrating Participatory Action Research With a Cluster Trial

**DOI:** 10.2188/jea.JE20170109

**Published:** 2019-02-05

**Authors:** Satoshi Seino, Akihiko Kitamura, Yui Tomine, Izumi Tanaka, Mariko Nishi, Kumiko Nonaka, Yu Nofuji, Miki Narita, Yu Taniguchi, Yuri Yokoyama, Hidenori Amano, Tomoko Ikeuchi, Yoshinori Fujiwara, Shoji Shinkai

**Affiliations:** 1 Research Team for Social Participation and Community Health, Tokyo Metropolitan Institute of Gerontology, Tokyo, Japan; 2 Japan Association for Development of Community Medicine, Tokyo, Japan; 3 Tokyo Metropolitan Institute of Gerontology, Tokyo, Japan

**Keywords:** frailty, community-wide intervention, exercise, nutrition, social participation

## Abstract

**Background:**

Preventing and reducing frailty is an important challenge for Japan in the next decade, especially in metropolitan areas. We launched a community-wide intervention trial (the Ota Genki Senior Project) in 2016 to develop effective community-based strategies for frailty prevention in metropolitan areas. This report describes the study design and baseline survey.

**Methods:**

This study is a community-wide intervention trial that integrates participatory action research into a cluster non-randomized controlled trial for adults aged 65 years or older living in Ota City, Tokyo. We allocated 3 of 18 districts to an intervention group and the other 15 to a control group. Using a mailed self-administered questionnaire, we conducted a baseline survey of 15,500 residents (8,000 and 7,500 in the intervention and control groups, respectively) from July through August 2016. In addition to socioeconomic status and lifestyle variables, we assessed frailty status (primary outcome) and physical, nutritional, and psychosocial variables (secondary outcomes). Based on the baseline findings, an intervention to improve outcomes will be implemented as participatory action research. Follow-up surveys will be conducted in the same manner as the baseline survey.

**Results:**

A total of 11,925 questionnaires were returned (76.9% response rate; 6,105 [76.3%] and 5,820 [77.6%] in the intervention and control groups, respectively), and 11,701 were included in the analysis (mean age, 74.3 [standard deviation, 5.5] years; 48.5% were men).

**Conclusions:**

This study is expected to contribute to development of a prototype of a community-wide frailty prevention strategy, especially in metropolitan areas in Japan. Trial registration: UMIN Clinical Trials Registry (UMIN000026515).

## INTRODUCTION

Frailty is a geriatric syndrome with multiple causes and contributors. It is characterized by diminished strength, endurance, and physiologic function, all of which increase the risks of increased dependence and death.^1^ Although frailty prevalence increases with age, especially in adults aged 75 or older,^2^ frailty can be prevented or reversed at an early stage. Therefore, preventing and reducing frailty is a major concern in geriatrics.

The estimated number of Japanese aged 75 years or older in 2025 (proportion of population, 18.1%) will be approximately 1.5 times that in 2010 (11.1%), because the postwar Baby-Boomer generation will reach age 75 by 2025.^3^^,^^4^ The phenomenon will be more remarkable in metropolitan areas.^4^ Thus, preventing and reducing frailty at the community level in metropolitan areas is an important challenge. We have conducted community-based interventions for frailty prevention in hilly and mountainous and suburban areas in Japan.^5^^–^^9^ However, to the best of our knowledge, no previous study has reported effective community-wide interventions or policies in Japanese metropolitan areas.

Physical exercise, nutritional education, and active social participation should be the main targets of frailty prevention interventions.^5^ Recent randomized controlled trials (RCTs) showed that a multifactorial intervention comprising these factors effectively reduced frailty and improved functional health.^8^^,^^10^^–^^12^ The next step in such research is to determine how elements of a multifactorial program can be translated into practice in a community. To verify effectiveness, it will be necessary to allocate communities rather than individuals.

On the basis of past studies, we launched the community-wide intervention trial for preventing and reducing frailty in Ota City, Tokyo (the Ota Genki Senior Project), in 2016. The aims of this study are to develop and examine the effectiveness of a social mechanism (ie, intervention content) that postpones frailty and to determine the requirements for transferring the intervention to other communities (transferability). Using this process, we aim to develop a prototype of a frailty prevention strategy for metropolitan areas in Japan. This report describes the study design and baseline profile of the participants.

## METHODS

### Study design

The Ota Genki Senior Project is a community-wide intervention study integrating participatory action research (PAR)^13^^,^^14^ into cluster non-RCTs for individuals aged 65 years or older living in Ota City, Tokyo, Japan. Although a multi-pronged approach is effective in postponing frailty,^8^^,^^10^^–^^12^ standardized interventional approaches are less successful at the community level because of inherent differences between communities, such as human and regional resources and organizational structure. Moreover, translating a “one-size-fits-all” intervention from one community to another may be difficult for the same reasons.

To overcome this problem, an implementation research design integrating PAR into RCTs was proposed as a new approach for studying interventions in healthcare settings.^15^ PAR is a design that brings together researchers and stakeholders (eg, residents, civil groups, professionals, company, and government) in a collaborative effort to address issues in specific systems.^13^^–^^16^ It is a collaborative, cyclical (analogous to “plan–do–check–act”), and reflective inquiry design that focuses on problem solving and understanding the effect of an intervention as part of the research process.^15^

We attempt to expand this design by integrating PAR into cluster non-RCTs. Hence, we can design interventional trials that are generalizable but have enough flexibility to be meaningful and are more likely to be successful locally.^15^ The trial is registered in the UMIN Clinical Trials Registry (UMIN000026515).

### Study setting

In response to a request from Ota City, we proposed and began a 3-year collaborative research project. In October 2011, we started work for 3 years on “Development of a Community-based Comprehensive System for Prevention of Frailty in Late Life.”^9^ The intent was to use high-quality evidence to create a frailty prevention theory and to use this theory for community planning aimed at postponing frailty through cooperation with community residents and government officials in Yabu City, Hyogo Prefecture (a hilly and mountainous area) and Hatoyama Town, Saitama Prefecture (a suburban area). On the basis of these premises, we hope to construct a model for frailty prevention in greater Tokyo.

Ota City is the southernmost of the 23 special wards of Tokyo. On August 1, 2016 it had a population of 716,645 (357,748 males and 358,897 females), 162,443 (71,481 men and 90,962 women) of whom were aged 65 years or older. The proportion of elders to the total population was 22.7%, which is slightly higher than the mean for the other special wards (21.1% as of January 2016).^17^ The area of Ota City (60.66 km^2^) is the largest of the special wards, although Tokyo International Airport accounts for approximately a quarter. The population density was 11,814 persons/km^2^ (habitable area: approximately 15,750 persons/km^2^).

### Allocation

**Figure 1.**
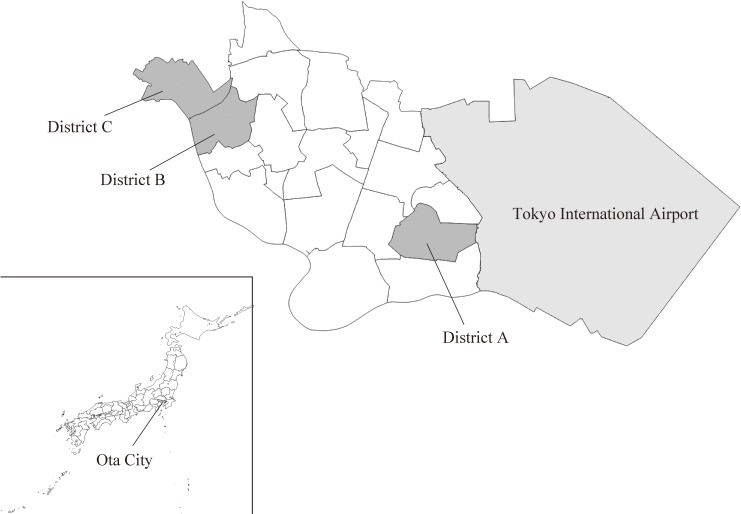
Geographical location of study areas (Ota City, Tokyo, Japan)

As shown in Figure 1, there are 18 administrative districts within Ota City, and the eligibility criterion for clusters applies to all districts. Normally, the intervention district should be selected randomly. However, the results of initial meetings with city employees and community diagnoses based on previous results of Ota City surveys of actual conditions indicated that east and west Ota City differed greatly in characteristics such as regional sources, participation rates for resident associations/neighborhood associations, and characteristics such as main cause of death. Therefore, we chose a non-randomized design and selected intervention districts from both east and west Ota City.

Next, arrangements were made at the Ward Office to select the intervention districts. District A (population, 38,301; elderly population, 8,797 [23.0%]), which is located in the east, and districts B and C (considered as one cluster; population, 47,183; elderly population, 10,914 [23.1%]), which are located in the west, were allocated to the intervention group; the other 15 districts were allocated to the control group. Statistical data on rates of long-term care insurance certification for Ota City, as of 2016, showed that the rates in districts A, B, and C were the second, fifth, and sixth highest, respectively, of the 18 districts, and we thus considered that these districts are suitable as intervention districts.

Districts B and C neighbor each other, and their elderly populations (district B, 5,514; district C, 5,400) are smaller than that of district A (elderly population, 8,797). In addition, discussions with the directors of the Community Support Service Centers revealed that there were few community resources, such as shopping centers, in district C, and that residents of district C also use shopping centers and sports clubs located in district B. Furthermore, the directors of Community Support Service Centers in both districts wanted to carry out joint regional activities between districts B and C in the future. Therefore, we decided to start the project by placing districts B and C in the same cluster.

### Overview of the intervention

**Figure 2.**
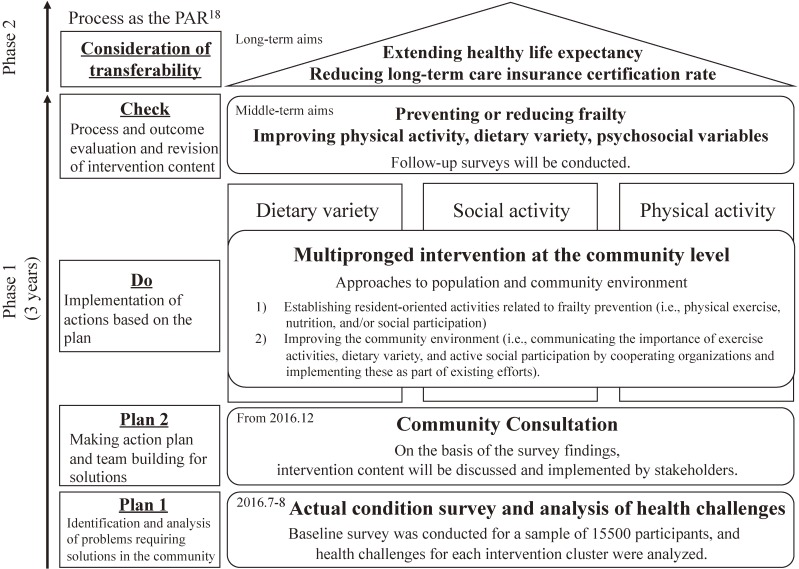
Research roadmap of the Ota Genki Senior Project. PAR, participatory action research.

Figure 2 shows the research roadmap of the Ota Genki Senior Project. The first step, conducting the baseline survey, was performed in July and August 2016. The data were used to analyze health challenges regarding exercise, nutrition, and/or social activity that should be addressed in each intervention district (“Plan 1”).

Second, to discuss and develop intervention content based on the baseline findings, a monthly “Community Consultation” is held in each intervention district. Members of the Community Consultation include stakeholders, such as residents, professionals (caseworkers, care managers, mental health welfare professionals, managerial dieticians, exercise instructors, and others), and members of Community Support Service Centers, social welfare corporations, employment service centers for older people, companies, research institutions, and government (city employees) (“Plan 2”).

Third, on the basis of the plan developed in the Community Consultation, interventions that focus on improving dietary variety and physical and social activities are implemented as PAR for each intervention district (“Do”). The intervention contents are 1) to establish resident-oriented activities related to frailty prevention (ie, physical exercise, nutrition, and/or social participation), and 2) to improve the community environment (ie, communicating the importance of exercise activities, dietary variety, and active social participation by cooperating organizations and implementing these as part of existing efforts).

Currently, in district A, regular group meals and walking groups are planned. A lecture series (lecture + exercise practice) is offered in all 10 neighborhood associations. Additionally, the “district A squat challenge” is being held using information communication equipment to disseminate the squatting exercise.

In district B, regular walking-pole groups are planned in all five neighborhood associations. Additionally, nutritional lectures and group meals are planned in various locations in the district, such as shopping malls. We are also developing software applications to visualize walking practices and daily food intakes.

In district C, in accordance with the concept of “community building that makes you want to go out,” the members are studying a plan to spread awareness of frailty prevention for participants, by holding regular events that include a walk around the district followed by a group meal.

Posters and recipes are created to raise awareness of the importance of dietary variety and are posted in shopping locations and public facilities in intervention districts. In the control group, usual health practices are continued.

Fourth, the intervention’s effects will be assessed by means of a 2-year follow-up survey, and the intervention content will be revised as needed (“Check”). Moreover, the requirements necessary for transfer of the intervention to other communities will be identified (“Consideration of Transferability”). In concrete terms, we will create a list of all intervention content implemented in the three intervention districts after the end of the 2-year follow-up survey. Using this list, we plan to host a core committee comprising ourselves, city employees, and directors of the Community Support Service Centers of each district to discuss implementation of the interventions in other districts.

Finally, we will examine whether the created social mechanism decreased the long-term care insurance certification rate and/or extended healthy life expectancy. These steps are based on the PAR framework.^18^

### Baseline and follow-up surveys

This baseline survey was conducted to collect baseline data for the intervention study, to clarify the current living conditions of older adults living in Ota City, and to serve as material for intervention content.

We used both stratified sampling of four groups classified by age group (65–74 and 75–84 years) and sex (men and women) and random sampling strategies for recruitment of study participants from the 18 districts. Individuals with long-term care insurance certification and those admitted to hospitals or residing in nursing homes were excluded. The target population for sampling was 15,500 residents (8,000 and 7,500 people in the intervention and control groups, respectively), which is equivalent to approximately one-tenth of the elderly population of Ota City. In the intervention group, 4,000 individuals (1,000 from each of the four stratified groups) from district A and 2,000 individuals (500 from each of the four stratified groups) each from districts B and C were recruited; thus, the questionnaire was distributed to approximately one out of every two residents. In the control group, 500 individuals (125 from each of the four stratified groups) from each of the other 15 districts were recruited.

On the basis of available data from the Yabu Cohort Study, the estimated prevalence of frailty at baseline was 27.5%,^7^ and the estimated intra-cluster correlation coefficient (ICC) was 0.0112 (ICC was unpublished). From these data, we assumed the prevalence of frailty in Ota City as 25%, with an ICC of 0.01. Sample size was calculated using the chi-square test at the individual level, a two-sided 5% significance level, and a power of 80% to detect a 6% difference in change in the prevalence of frailty between the intervention and control groups, without considering the design effect (DE) by cluster trial.^19^ Thus, samples of 748 persons were obtained for the intervention and control group (total: 1,496). When we assume a final sample with a 50% response rate at baseline, the estimated DE is 5.29. The product of the obtained sample size of 748 in the intervention and control groups and estimated DE of 5.29 is 3,957. Therefore, we considered that recruiting 8,000 participants in the intervention group and 7,500 participants in the control group would retain the expected statistical power throughout follow-up.

After conducting the baseline survey, a 2-year follow-up questionnaire survey is planned. After that, follow-up surveys will be repeated every 3 years. Follow-up questionnaires will be mailed to respondents to the baseline survey. To improve assessment of individual-level and community-level effects, we will add an additional sample of randomly extracted adults of the same sex and age group as those who dropped out at each follow-up survey.

As in many previous studies,^20^^–^^22^ it may be difficult to produce outcome changes in only 2 years, and long-term follow-up may be required. We plan to repeat follow-up surveys every 3 years after the 2-year follow-up. Moreover, we plan to use a stepped-wedge design^23^ to expand the intervention areas within the 18 districts, apart from districts A, B, and C. Differences between the first intervention group (districts A, B, and C), districts included in the following intervention areas, and control districts will be examined by every follow-up survey. Furthermore, the previous logic model suggests that change would be induced by the intervention in the following order: awareness, knowledge, belief, intention, and finally action,^20^ and we will identify the stage we have reached at each follow-up survey.

Finally, mortality and long-term care insurance certification after the baseline survey are expected to be investigated in future exploratory studies.

### Outcomes

As shown in Table 1, outcome measures include frailty status (primary outcome) and physical and psychosocial variables and dietary variety (secondary outcomes), in addition to socioeconomic status and lifestyle variables. Procedures for assessing the primary and secondary outcome measures and additional measures are detailed in eMaterial 1.

**Table 1.** Summary of items surveyed in the Ota Genki Senior Project at baseline, 2016

**Primary outcome measures**
Check-List 15 and frailty status
**Secondary outcome measures**
Physical activity and physical function
Exercise habits
International Physical Activity Questionnaire Short-Form
Motor Fitness Scale
Mobility limitations
Dietary variety
Dietary Variety Score
Food Frequency Score
Psychosocial function
Self-rated health
Five-item version of the Geriatric Depression Scale
WHO-5 Well-Being Index
Frequency of outing
Social isolation
Cognitive social capital (trust in neighbors, etc)
Structural social capital (social participation, etc)
**Additional measures**
Age
Sex
Living arrangement
Marital status
Years of residence in neighborhood area
Socioeconomic status (education, household income, etc)
Employment status
History of physician-diagnosed diseases and chronic musculoskeletal pain
Body mass index (self-rated height and weight)
Drinking and smoking
Sleep duration, difficulty falling asleep, and quality of sleep
Pet breeding situation
Information and communication technology use
Tokyo Metropolitan Institute of Gerontology Index of Competence
Number of meals
Eating alone
Level of happiness
Social network, social support, and neighbor relationships

WHO, World Health Organization.

### Primary outcome measures

The primary outcomes are Check-List 15 (CL15) score and frailty status (frailty defined as a CL15 score ≥4).^5^^,^^24^^,^^25^ CL15 score was treated as both a continuous and dichotomous (prevalence of frailty) variable in the analysis.

### Secondary outcome measures

#### Physical activity and physical function

Engaging in any exercise and muscle-strengthening activities 1 or more days/week and engaging in 150 minutes/week or more of walking were evaluated.^20^^,^^26^^–^^28^ Physical function was assessed using the Motor Fitness Scale.^29^^,^^30^ Mobility limitations were identified through self-reported difficulty in walking one-quarter of a mile (0.4 km) or climbing 10 steps without resting.^31^^,^^32^

#### Dietary variety

Dietary Variety Score (DVS, range 0–10)^33^^,^^34^ and Food Frequency Score (FFS, range 0–30)^34^ were assessed with a self-administered questionnaire. DVS was treated as both a continuous and dichotomous (score of ≥4) variable.

#### Psychosocial function

Self-rated health, depressive mood, well-being, frequency of outing, social isolation, and cognitive and structural social capital were determined by analyzing responses to a commonly used self-administered questionnaire.^35^^–^^39^

### Ethical considerations

The study protocol was developed in accordance with the guidelines proposed in the Declaration of Helsinki and was approved by the Ethics Committee of the Tokyo Metropolitan Institute of Gerontology (approved June 1, 2016). All participants gave informed consent. A statement attached to the questionnaire explained the purpose of the study, the voluntary nature of participation, and a promise of anonymity in the analysis. Returning the questionnaire was viewed as consent to participate in the study.

### Statistical analyses

All data in the text and tables are presented as means (standard deviations) or proportions. We used the unpaired *t* test, Mann-Whitney *U* test, or chi-square test to compare baseline characteristics between groups. Moreover, to compare district A, districts B and C with the control, we used analysis of variance, the Kruskal-Wallis test, or chi-square test. Post-hoc multiple-comparison testing with Bonferroni adjustment was performed for primary and secondary outcome measures. We calculated the ICCs for primary and secondary outcome measures as follows:

ICC=(BMS−WMS)/(BMS+[K−1]WMS),

where BMS is the between-cluster mean square, WMS is the within-cluster mean square, and K is the average number of respondents per cluster.^19^^,^^20^ An α of 0.05 was considered to indicate statistical significance, and all statistical analyses were performed using Stata 14.0 (Stata Corp, College Station, TX, USA).

As primary analyses, we will use generalized linear mixed-effects models (GLMM) to compare changes in all outcomes between the intervention group (3 districts) and control group (15 districts). Each outcome will be defined as a dependent variable. Group, time (baseline and follow-up), and their interaction will be defined as fixed factors, and the districts where respondents live will be defined as a random factor. Sex, age, body mass index, chronic disease, socioeconomic status, and lifestyle at baseline, and the baseline value of each outcome will be defined as covariates. The intervention effect and its 95% confidence interval will be calculated as an estimate of the mean differences in changes between groups, after controlling for covariates.

As secondary analyses, we will use a GLMM to compare each intervention district to the control group for primary and secondary outcomes, in the same manner. Furthermore, subgroup analyses will be used to detect significant differences in the intervention effect, after stratification by sex and/or age group (65–74 and 75–84 years).

## RESULTS

**Figure 3.**
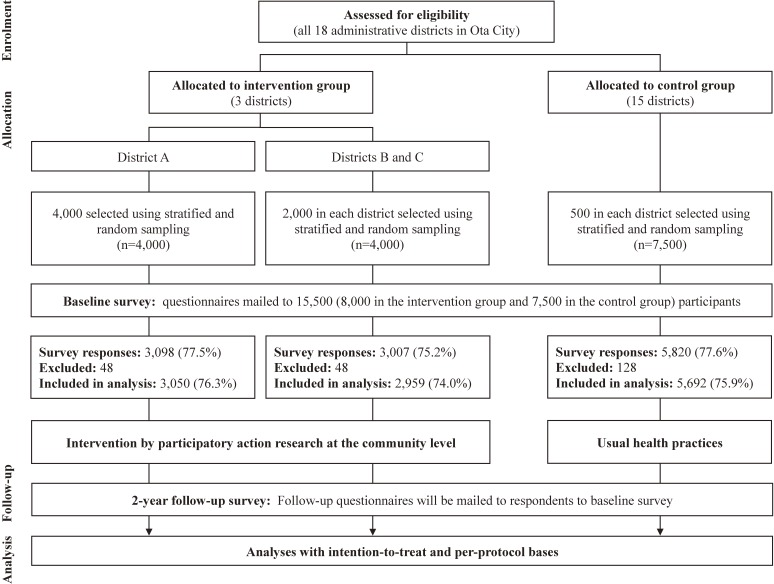
Flow diagram of progress in the Ota Genki Senior Project

Figure 3 shows a flow diagram of the study’s progress. Of the 15,500 questionnaires distributed, 11,925 were returned (76.9% response rate; 6,105 [76.3%] and 5,820 [77.6%] of the intervention and control groups, respectively). After excluding 79 questionnaires from respondents who did not actually live in the city, 38 questionnaires that were almost or completely blank, 19 questionnaires with missing identification labels, 22 questionnaires that were completed by someone other than the participant, and 66 questionnaires from hospital inpatients and nursing home residents (total 224), 11,701 were ultimately identified as the study population and included in the analysis (75.5% valid response rate; 6,009 [75.1%] and 5,692 [75.9%] of the intervention and control groups, respectively).

Table 2 shows the baseline characteristics of the study population. Compared with participants in the control group, those in the intervention group were less likely to be living alone, had less current smoking, knee pain, depressive mood, and social isolation, were more likely to be married and have social participation more than once a week, and had higher educational level, equivalent income, and DVS and FFS scores. The multiple-comparison test showed that frailty status and almost all secondary outcome measures were significantly worse in district A and better in districts B and C than in the control group. No significant difference was observed in any other measure.

**Table 2.** Baseline characteristics of participants selected with stratified and random sampling, by age and sex (Ota Genki Senior Project in 2016)

Variables	Intervention group						Control group		*P*-value		ICC^d^
	
	All		District A		Districts B and C				Intervention vs control^b^	A vs B & C vs control^c^	
Number of clusters	3		1		2		15				
Number of residents	85,484		38,301		47,183		631,161				
Number of residents aged 65 or older	19,711		8,797		10,914		142,732				
Number of analyzed participants (eligible response rate)	6,009	(75.1)	3,050	(76.3)	2,959	(74.0)	5,692	(75.9)			
Age, years, mean (SD)	74.3	(5.4)	74.1	(5.3)	74.4	(5.5)	74.3	(5.5)	0.93	0.08	
65–74, *n* (%)	2,848	(47.4)	1,468	(48.1)	1,380	(46.6)	2,669	(46.9)	0.58	0.44	
75–84, *n* (%)	3,161	(52.6)	1,582	(51.9)	1,579	(53.4)	3,023	(53.1)			
Male, *n* (%)	2,902	(48.3)	1,465	(48.0)	1,437	(48.6)	2,768	(48.6)	0.72	0.86	
Living alone, *n* (%)	1,136	(19.3)	628	(21.1)	508	(17.5)	1,242	(22.4)	<0.001	<0.001	
Marital status, *n* (%)									<0.001	<0.001	
Married	4,032	(68.8)	1,979	(66.9)	2,053	(70.7)	3,598	(64.8)			
Widowed or Divorced	1,460	(24.9)	787	(26.6)	673	(23.2)	1,494	(26.9)			
Never married	367	(6.3)	190	(6.4)	177	(6.1)	460	(8.3)			
Education, *n* (%)									<0.001	<0.001	
Junior high school graduate	1,496	(24.9)	1,207	(39.6)	289	(9.8)	1,414	(24.8)			
High school graduate	2,122	(35.3)	1,207	(39.6)	915	(30.9)	2,287	(40.2)			
Junior college/vocational college graduate	691	(11.5)	231	(7.6)	460	(15.5)	637	(11.2)			
College/graduate school graduate	1,457	(24.2)	238	(7.8)	1,219	(41.2)	1,158	(20.3)			
Other/unknown	243	(4.0)	167	(5.5)	76	(2.6)	196	(3.4)			
Equivalent income, *n* (%)									<0.001	<0.001	
≥4.0 million yen	1,191	(19.8)	344	(11.3)	847	(28.6)	914	(16.1)			
2.5–3.99 million yen	1,385	(23.0)	613	(20.1)	772	(26.1)	1,401	(24.6)			
1.0–2.49 million yen	1,811	(30.1)	1,171	(38.4)	640	(21.6)	1,819	(32.0)			
<1.0 million yen	419	(7.0)	290	(9.5)	129	(4.4)	390	(6.9)			
Unknown	1,203	(20.0)	632	(20.7)	571	(19.3)	1,168	(20.5)			
Alcohol drinking status, *n* (%)									0.24	<0.001	
Current	3,288	(55.4)	1,599	(53.2)	1,689	(57.7)	3,037	(54.0)			
Never or former	2,645	(44.5)	1,408	(46.8)	1,237	(42.3)	2,584	(46.0)			
Smoking status, *n* (%)									0.036	<0.001	
Current	702	(11.9)	412	(13.9)	290	(9.9)	748	(13.4)			
Never or former	5,192	(88.0)	2,562	(86.2)	2,630	(90.0)	4,823	(86.6)			
Body mass index (kg/m^2^), mean (SD)	22.7	(3.2)	23.1	(3.2)	22.4	(3.1)	22.7	(3.2)	0.35	<0.001	
Number of chronic diseases, *n* (%)^a^									0.78	0.59	
0	1,391	(25.7)	675	(24.8)	716	(26.6)	1,294	(25.5)			
1	1,767	(32.6)	898	(33.0)	869	(32.3)	1,691	(33.3)			
2^+^	2,257	(41.7)	1,150	(42.2)	1,107	(41.1)	2,097	(41.3)			
Musculoskeletal pain, *n* (%)											
Shoulder	1,213	(22.1)	654	(23.4)	559	(20.7)	1,198	(23.1)	0.22	0.025	
Low back	2,228	(39.9)	1,186	(42.0)	1,042	(37.8)	2,095	(39.8)	0.90	0.007	
Knee	1,762	(31.8)	998	(35.4)	764	(28.0)	1,775	(34.0)	0.016	<0.001	
**Primary outcome measures**											
Frailty (CL15 score ≥4), *n* (%)	1,248	(23.0)	726	(27.0)	522	(19.1)^*^	1,243	(24.1)^*,**^	0.18	<0.001	0.0072
CL15 score (0–15), mean (SD)	2.3	(2.1)	2.5	(2.1)	2.1	(2.0)^*^	2.4	(2.1)^*,**^	0.32	<0.001	0.0085
**Secondary outcome measures**											
Physical activity and physical function											
Engaging in any exercise more than once a week, *n* (%)	4,341	(73.9)	2,061	(69.5)	2,280	(78.3)^*^	4,095	(73.9)^*,**^	0.98	<0.001	0.0066
Engaging in ≥150 minutes/week of walking, *n* (%)	3,045	(70.2)	1,389	(66.6)	1,656	(73.4)^*^	2,958	(71.9)^*^	0.08	<0.001	0.0051
Engaging in muscle-strengthening activities more than once a week, *n* (%)	533	(9.1)	200	(6.7)	333	(11.4)^*^	511	(9.2)^*,**^	0.78	<0.001	0.0036
Motor Fitness Scale (0–14), mean (SD)	10.6	(3.3)	10.3	(3.3)	10.9	(3.2)^*^	10.5	(3.3)^*,**^	0.32	<0.001	0.0081
Low physical function (score ≤11 for men, ≤9 for women), *n* (%)	2,149	(39.5)	1,184	(43.5)	965	(35.5)^*^	2,086	(40.6)^*,**^	0.25	<0.001	0.0061
Mobility limitation, *n* (%)	1,742	(29.4)	1,051	(35.1)	691	(23.6)^*^	1,697	(30.3)^*,**^	0.29	<0.001	0.0116
Dietary intake											
Dietary Variety Score (0–10), mean (SD)	3.2	(2.2)	2.9	(2.2)	3.5	(2.2)^*^	3.1	(2.2)^*,**^	0.040	<0.001	0.0175
Score ≥4, *n* (%)	2,237	(41.5)	937	(35.3)	1,300	(47.5)^*^	2,009	(39.3)^*,**^	0.021	<0.001	0.0123
Food Frequency Score (0–30), mean (SD)	18.0	(5.2)	17.2	(5.3)	18.8	(5.0)^*^	17.7	(5.2)^*,**^	0.002	<0.001	0.0185
Psychosocial function											
Self-rated health (excellent to good), *n* (%)	4,514	(80.6)	2,160	(76.7)	2,354	(84.6)^*^	4,298	(80.9)^*,**^	0.69	<0.001	0.0083
Depressive mood (GDS-5 score ≥2), *n* (%)	1,877	(33.7)	1,070	(38.5)	807	(29.0)^*^	1,889	(35.8)^**^	0.021	<0.001	0.0115
Well-being (WHO-5 Well-Being Index: 0–25), mean (SD)	15.3	(6.0)	14.5	(6.3)	16.1	(5.6)^*^	15.2	(6.0)^*,**^	0.37	<0.001	0.0134
Outing more than once a day, *n* (%)	4,366	(73.8)	2,220	(74.2)	2,146	(73.3)	4,202	(75.0)	0.14	0.25	0.0009
Social isolation, *n* (%)	1,632	(27.8)	951	(32.1)	681	(23.4)^*^	1,664	(29.9)^**^	0.013	<0.001	0.0060
Trust in neighbors, (agree or tend to agree), *n* (%)	4,414	(77.5)	2,113	(74.0)	2,301	(81.0)^*^	4,129	(76.3)^*,**^	0.16	<0.001	0.0055
Social participation more than once a month, *n* (%)	2,387	(45.5)	996	(38.1)	1,391	(52.8)^*^	2,141	(43.3)^*,**^	0.027	<0.001	0.0163

CL15, Check-List 15; GDS-5, 5-item version of the Geriatric Depression Scale; ICC, intracluster correlation coefficient; SD, standard deviation; WHO, World Health Organization.

^a^Sum of the presence of hypertension, hyperlipidemia, cardiovascular disease, cerebrovascular disease, and diabetes mellitus.

^b^Comparison between intervention and control groups; chi-square test used for categorical variables and unpaired *t* or Mann-Whitney *U* test used for continuous variables.

^c^Comparison between district A, districts B and C, and control groups; chi-square test used for categorical variables and analysis of variance or Kruskal-Wallis test used for continuous variables.

Post-hoc multiple-comparison testing with Bonferroni adjustment was performed for primary and secondary outcome measures: ^*^P < 0.017 versus district A; ^**^P < 0.017 versus districts B and C.

^d^ICCs of primary and secondary outcome measures were calculated as follows: ICC = (BMS − WMS)/(BMS + [K − 1]WMS), where BMS is the between-cluster mean square, WMS is the within-cluster mean square, and K is the average number of respondents per cluster.

The results of analysis of the additional measures are shown in eTable 1. As compared with participants in the control group, those in the intervention group were more likely to eat with others and to be working and had more interactions with neighbors. No significant difference was observed in any other measure.

## DISCUSSION

We described the study design and baseline characteristics of participants in the present study, which was launched in 2016. To the best of our knowledge, this is the first intervention study for community-wide frailty prevention in a metropolitan area.

Dietary variety and some variables related to psychosocial and socioeconomic status were better for participants in the intervention group than for those in the control group. Further multiple-comparison testing showed that socioeconomic status, frailty status, and physical, nutritional, and psychosocial variables were notably worse in district A than in districts B and C and the control group, regardless of age. For this reason, district A is positioned as a “typical model area” for solving health challenges. Because interaction with neighbors is active in district A (eTable 1), the word-of-mouth information dissemination strategy using this feature was considered effective. Based on proportionate universalism (that is, applying a universal intervention with increased intensity in groups with greater need),^40^^,^^41^ intensive intervention is needed for district A in particular within the intervention group.

Socioeconomic status, frailty status, and secondary outcome measures were better in districts B and C than in the control group, regardless of age. In addition, the utilization rate of information and communication technology (ICT), such as smartphones, computers, and internet was prohibitively high (eTable 1). In the next decade, the affinity of the elderly for ICT is expected to progressively increase. Therefore, we positioned districts B and C as a model area for development of ICT equipment and applications that can be used for frailty prevention (promoting physical exercise, diverse food intake, and active social participation).

Continuation of community-wide intervention studies and resident-oriented activities will require an annual budget of more than 10 million yen, especially when both the intervention and follow-up surveys are conducted on this scale. However, implementation of all the content of this research in other communities is labor- and cost-intensive and may not be realistic. Therefore, we will create a project content list (eg, regular walking group, group meals, and/or printing for the creation and distribution of pamphlets) that will explicitly indicate the processes implemented in them and how much funding was needed for each component. Using this list, we believe it will be easier for other communities to select and implement the components of this study.

This is an effectiveness study that attempts to disseminate and implement its findings,^8^ the effects of which were examined in a RCT (efficacy study). Combining PAR and cluster non-RCTs, as in the present study, increases the ability to translate research findings into general practice in other communities. The process and results of this study can contribute to future dissemination and implementation research and to a Community-based Integrated Care System,^42^ which is promoted in Japan.

Our study has some limitations and concerns. First, the self-administered questionnaire used for outcome measurement may be participant to recall bias. Second, complete prevention of potential contamination is difficult because this study is performed as part of an administrative policy. Similarly, public relations at public facilities or shopping malls in intervention districts will be effective, but it is difficult to prevent contamination as new people enter the intervention district. Third, substantial attrition during the follow-up surveys and a large difference between the intervention and control groups will lead to attrition bias and loss of statistical power. Because the response rate will be increased with established methods^43^ (eg, sending postcard reminders to non-responders, mayoral announcements, establishing a mechanism for inquiries within the district for questions, and requesting cooperation from community readers), considerable effort will be required in order to increase the response rate. Finally, this study does not have a randomized design. However, a non-randomized design can contribute to developing evidence-based public health policies^44^ because its feasibility is high.

In conclusion, the Ota Genki Senior Project was launched in 2016 to develop and evaluate the effectiveness of a social mechanism for postponing frailty. The intervention will be carried out using PAR, in accordance with baseline survey results. Follow-up questionnaire surveys are planned. This study is expected to contribute to development of a prototype of a community-wide frailty prevention strategy in metropolitan areas in Japan.

## APPENDIX A. SUPPLEMENTARY DATA

eMaterial 1. Procedures for assessing primary, secondary, and additional measures

eTable 1. Baseline characteristics of participants (measures not included in Table 2)
