# Supplementary material for: A Community-Wide Intervention Trial for Preventing and Reducing Frailty Among Older Adults Living in Metropolitan Areas: Design and Baseline Survey for a Study Integrating Participatory Action Research With a Cluster Trial
Source: J Epidemiol. 2019 Feb 5;29(2):73–81. doi: 10.2188/jea.JE20170109 (PMC6336723; doi:10.2188/jea.JE20170109)
Supplement: Supplementary file 1 [file je-29-073-s001.pdf]

eMaterial 1. Procedures for assessing primary, secondary, and additional measures

Primary outcome measures

The primary outcomes were Check-List 15 (CL15) score and frailty status (frailty defined as a CL15 score ≥4).[5, 24, 25] The CL15 comprises 15 self-administered items, including the dimensions of homeboundness, falling, and lower nutrition. The index score ranges from 0 to 15, and a higher score indicates a greater likelihood of frailty. Shinkai et al.[25] reported that a cut-off score of 3/4 discriminated Fried's frailty[45] from non-frailty with a sensitivity of 70.0% and specificity of 89.3%. They confirmed that older persons with CL15 scores of 4 or higher had a significantly higher risk for developing future adverse health outcomes, such as disabilities related to activities of daily living or mortality, compared with those who had CL15 scores of 3 or lower.[25] CL15 score was treated as both a continuous and dichotomous (prevalence of frailty) variable in the analysis.

Secondary outcome measures

Physical activity and physical function

Engaging in any exercise and muscle-strengthening activities 1 or more days/week and

150 minutes/week or more of walking were evaluated.[20, 26] We asked about performing exercise more than once a week by multiple answers. Recreational walking and walking for transport were both considered engagement in walking. We asked about the number of days per week and the mean number of minutes walked per day, which are part of the International Physical Activity Questionnaire Short-Form[27, 28] and calculated the weekly total minutes of walking time.

Physical function was assessed by using the Motor Fitness Scale.[29, 30] The responses to each item in this 14-item index of physical tasks are “yes” (able to perform), for 1 point, and “no” (unable to perform), for 0 points. The total score ranges from 0 to 14. Lower scores indicate lower physical function, and a total score of ≤11 for men or ≤9 for women is defined as low physical function.[30] Mobility limitation was defined as self-reported difficulty in walking one-quarter of a mile (0.4 km) or climbing 10 steps without resting.[31] The response options were “no difficulty,” “some difficulty,” or “inability” to perform. Those who reported at least some difficulty performing these activities were considered to have limited mobility.[32]

Dietary variety

We assessed the frequency of food intake and dietary variety by using a

self-administered questionnaire.[33, 34] Over a period of 1 week, participants were asked about their frequency of consumption for each of the 10 main food groups in the Japanese diet (meat, fish/shellfish, eggs, milk, soybean products, green/yellow vegetables, potatoes, fruit, seaweed, and fats/oils). There were four responses for frequency of food intake for each food group: almost daily (3 points), 3 or 4 days a week (2 points), 1 or 2 days a week (1 point), and hardly ever (0 points). Food Frequency Score (FFS)[34] was calculated as the sum of scores for each of the 10 food groups (range 0−30), and Dietary Variety Score (DVS) as the sum of the number of times each participant answered "almost daily" for each food group (range 0−10).[33] DVS was treated as both a continuous and dichotomous (score of ≥4) variable.

Social activity and psychosocial function

Self-rated health, depressive mood, well-being, frequency of outing, social isolation, and cognitive and structural social capital were evaluated by analyzing responses to a commonly used self-administered questionnaire. Self-rated health was assessed with the question, "How would you rate your current overall health?" The response options were "excellent," "good," "fair," and "poor." The 5-item Geriatric Depression Scale (GDS-5)[35, 36] and World Health Organization-Five (WHO-5) Well-Being Index[37, 38] were

used to assess depressive mood and well-being, respectively. We asked about frequencies of outing and contact with family/relatives and friends, apart from cohabiting family members. Contact with others (apart from cohabiting family members) less than once a week was defined as social isolation.[39] Cognitive social capital included trust in neighbors. The response options were “agree,” “tend to agree,” “tend to disagree,” and “disagree.” Structural social capital included frequency of social participation. Participation in any of the following activities or groups more than once a month was defined as the presence of social participation: volunteering, civic action, and nonprofit organizations; sports groups; hobby and learning groups; senior citizen clubs; neighborhood associations; and others.

Additional measures

We obtained information on age, sex, family composition (not alone or alone), marital status (married, widowed, divorced, or never married), education (junior high school graduate, high school graduate, junior college/vocational college graduate, college/graduate school graduate, or other/unknown), equivalent income (<1.0, 1.0–2.49, 2.5–3.99, ≥4.0 million yen), alcohol drinking and smoking status (current, former, or never), body mass index (self-rated height and weight), history of physician-diagnosed

diseases (hypertension, hyperlipidemia, cardiovascular disease, cerebrovascular disease, diabetes mellitus, and/or others), musculoskeletal pain (shoulder, low back, and/or knee), sleep duration, quality of sleep (excellent, good, fair, or poor), number of meals (n/day), eating status (eat with others or eat alone),[46] neighbor relationships (visiting each other, standing and chatting, no more than an exchange of greetings, or none), employment status (yes or no), information and communication technology use (any, smartphone, mobile phone, computer, internet, or none), Pet ownership (current, former, or never), subjective happiness (happy, rather happy, rather unhappy, or unhappy), and Tokyo Metropolitan Institute of Gerontology Index of Competence.[47]

eTable 1. Baseline characteristics of participants (measures not included in Table 2)

Variables	Intervention group			Control group	*P*-value	
	All	District A	Districts B and C		Intervention vs. control [a]	A vs. B & C vs. control [b]
Sleep duration, minutes, mean (SD)	397.3 (73.4)	396.9 (77.5)	397.8 (68.9)	395.6 (73.9)	0.20	0.40
Sleep duration categories, n (%)					0.52	
<6 hours	1143 (19.4)	614 (20.7)	529 (18.1)	1099 (19.7)		
6-8.9 hours	4509 (76.5)	2206 (74.4)	2303 (78.6)	4226 (75.8)		
≥9 hours	242 (4.1)	144 (4.9)	98 (3.3)	250 (4.5)		
Quality of sleep, excellent to good, n (%)	4286 (72.1)	2110 (70.2)	2176 (74.1)	3996 (71.2)	0.26	0.002
Number of meals, n/day, n (%)					0.25	0.006
3	5013 (84.0)	2495 (82.5)	2518 (85.6)	4709 (83.5)		
2	906 (15.2)	509 (16.8)	397 (13.5)	867 (15.4)		
1	24 (0.4)	12 (0.4)	12 (0.4)	36 (0.6)		
Others	22 (0.4)	8 (0.3)	14 (0.5)	27 (0.5)		
Eating status, n (%)					0.001	<0.001
Eat with others	5042 (84.4)	2478 (81.8)	2564 (87.0)	4647 (82.1)		
Eat alone	932 (15.6)	550 (18.2)	382 (13.0)	1010 (17.9)		
Neighbor relationships, n (%)					0.015	<0.001
Visiting each other	1413 (23.7)	775 (25.7)	638 (21.7)	1293 (22.7)		
Standing and chatting	2241 (37.7)	1113 (36.9)	1128 (38.4)	2092 (37.1)		
No more than an exchange of greetings	1939 (32.6)	941 (31.2)	998 (34.0)	1837 (32.5)		
None	359 (6.0)	184 (6.1)	175 (6.0)	424 (7.5)		
Employment, n (%)	2051 (35.6)	1075 (37.3)	976 (34.0)	1819 (33.5)	0.019	0.002
Information and communication technology use, n (%)						
Any	4620 (78.5)	2150 (72.6)	2470 (84.6)	4329 (77.7)	0.27	<0.001
Smartphone	1046 (17.8)	397 (13.4)	649 (22.2)	913 (16.4)	0.047	<0.001
Mobile phone (other than smartphone)	3471 (59.0)	1717 (58.0)	1754 (60.0)	3302 (59.2)	0.79	0.26
Computer	1792 (30.5)	528 (17.8)	1264 (43.3)	1590 (28.5)	0.024	<0.001
Internet	1403 (23.8)	389 (13.1)	1014 (34.7)	1270 (22.8)	0.18	<0.001
Pet ownership, n (%)					0.19	0.002
Current	957 (16.4)	437 (14.9)	520 (18.0)	856 (15.5)		
Never or former	4877 (83.6)	2501 (85.1)	2376 (82.0)	4669 (84.5)		
Subjective happiness, happy to rather happy, n (%)	5497 (93.5)	2724 (91.8)	2773 (95.3)	5198 (93.3)	0.67	<0.001
TMIG-IC (score, 0-13), mean (SD)	11.4 (1.9)	11.1 (2.1)	11.7 (1.7)	11.3 (1.9)	0.06	<0.001

SD, standard deviation; TMIG-IC, Tokyo Metropolitan Institute of Gerontology Index of Competence.

[a]Comparison between intervention and control groups; chi-square test used for categorical variables and unpaired *t* or Mann-Whitney *U* test used for continuous variables.

[b]Comparison between district A, districts B and C, and control groups; chi-square test used for categorical variables and analysis of variance or Kruskal-Wallis test used for continuous variables.
